# Nowcasting and monitoring SDG 8

**DOI:** 10.1007/s10663-022-09533-0

**Published:** 2022-03-02

**Authors:** Sandra Bilek-Steindl, Thomas Url

**Affiliations:** Austrian Institute of Economic Research (WIFO), Vienna, Austria

**Keywords:** Dynamic factor models, Nowcasting, Sustainable Development Goals, EU SDG indicators

## Abstract

We propose a nowcasting approach for indicators assigned to the Sustainable Development Goal (SDG) 8, calling for decent work and economic growth. The nowcasts of SDG indicators are based on dynamic factor models. In this mixed frequency framework, we exploit information from a comprehensive set of quarterly data to nowcast annually observed SDG indicators. For the model selection and specification search we evaluate the nowcast properties of the models based on a pseudo real-time data set. More recent information on SDGs can disclose a possible deviation from the desired path at an early stage. As an example, we present nowcasts for SDG objectives in Austria for the year 2020. The design of our assessment follows the method and quantitative rules suggested by Eurostat. SDG 8 indicators are highly related to the underlying economic situation and the effects of the COVID-19 pandemic are clearly visible in the results for 2020.

## Introduction

A further improvement of well-being metrics was one of the concluding recommendations in Stiglitz et al. (2018). Their reasoning concentrated on the need for additional information in a comprehensive political decision-making process aiming at a more general concept of sustainable development and progress. Particularly, the identification of top priorities, the subsequent monitoring of programme implementations, and the need for evaluation require accurate and timely published statistical indicators of well-being.

Already in 2005, measuring progress towards sustainable development was an issue for the European Commission (Eurostat, 2005). In 2009 the European Commission published a communication (COM (2009) 433) on initiating and constructing additional indicators going beyond GDP. Therein the European Commission stressed that if an indicator can summarise the relevant information about a goal with a single figure, it can be applied as a useful communication tool. Suitable indicators can initiate a policy debate and—for goals with already established policy targets—they provide the public with an impression about progress towards targets. Consequently, Eurostat further developed a set of indicators and published monitoring reports on the EU sustainability development strategy (e.g. Eurostat 2015). In 2017 the set was adjusted to 17 groups of indicators, each representing one of the Sustainable Development Goals (SDGs) adopted in 2015 by the United Nations within the framework of the 2030 Agenda for Sustainable Development. The 17 SDGs comprise economical, ecological and social aspects. The progress towards the goals is monitored by Eurostat in an EU context at an annual base (e.g. Eurostat 2020).

SDG 8 calls for inclusive and sustainable economic growth, full and productive employment and decent work for all. Sustainable and inclusive economic growth is the result of successful integration of economic, social, and environmental targets relating to full and productive employment and decent work (ILO 2019). UN (2020) also points at the close relation between inclusive growth and improved living standards. In this sense, UN (2020) is speaking about decent work as the opportunity for everybody to engage in work that is productive and offers a fair income, security at the workplace and social protection for families, better prospects for personal development and social integration. Adequate education providing youth with necessary skills for the labour market is also a fundamental issue in this respect.

SDG 8 covers conventional economic policy goals like GDP growth and employment for which governments established agencies compiling the relevant data long ago. Since many years, national and international research institutes engage in forecasting economic activity to provide guidance for economic policy. The goals summarised in SDG 8, however, go also beyond conventional economic indicators, extending them to inclusive and sustainable growth and decent work for all. Indicators reflecting a broader view on the labour market, like the “inactive population due to caring responsibilities” or covering information on “people killed in accidents at work” or the “work at-risk-of-poverty rate” now complement traditional economic variables. Some of these indicators, however, are published with considerable time lag and there is not yet an established regular forecasting process covering them. The lack of timely information became obvious in the light of the COVID-19 pandemic, as the lockdown measures, necessary to confine the spread of the virus, did not only create a steep decline in economic activity but also had consequences on the amount of caring responsibilities within families, when schools switched from class room to digital teaching. In addition to the negative consequences for employment, the sharp economic downturn also weighed on working income due to the reduction in working hours or the partial income loss associated with short-time work schemes. The strong interaction between labour market data and the disposable income of private households creates synergies between the nowcasting of key and multipurpose indicators in SDG 8. Whereas the rapid design and implementation of fiscal policy measures has been supported by nowcast information based on very high-frequency data (daily or weekly), up-to-date indicators highlighting beyond GDP goals have only been partially available. Especially ecological indicators are published with a delay, which impedes timely monitoring and quick policy intervention.

In this paper, we propose to transfer the application of the nowcasting technology from conventional economic variables to beyond GDP indicators. This provides decision makers and participants in policy discussions with an up-to-date information set to assess progress towards targets or to design countervailing policy measures in case of an expected deviation from target, similar to discretionary stabilisation policy in the case of business cycle fluctuations.

On the methodological side, over the past 2 decades the growing need for timely information in the decision-making process was met by the development of nowcasting models. Banbura et al. (2013) provide an overview on how to use data published at monthly or higher frequencies in a nowcasting model for quarterly GDP within the restrictions imposed by the publishing calendar of statistical agencies. In this paper, we suggest using nowcasting tools to supplement conventional short-term forecasts of economic activity during the current year with nowcasts of related SDG 8 indicators. Bierbaumer-Polly et al. (2019) develop an SDG nowcasting system for several key indicators assigned to ten out of the 17 SDG goals published by Eurostat and assess the progress towards SDGs in Austria for the year 2019. Here we will extent this work towards all key indicators as well as multi-purpose indicators assigned to SDG 8. For the nowcasting we use dynamic factor models, either based on a narrow set of variables following the extension of the Stock and Watson (1991) approach as suggested by Mariano and Murasawa (2003). Alternatively, we use a broad set of variables and apply the two-step estimator by Doz et al. (2011). We use quarterly variables ranging from national labour market and income data to quarterly national accounts data, industrial production, business cycle surveys, foreign trade statistics, data on care allowance recipients and demographic data sources.

The formal evaluation of the 2020’s SDGs by Eurostat has been published in June 2021 (Eurostat 2021). This features a publication lag of 6 months for most series. As regards to SDG 8 there is a lag of up to more than 1 year for the indicator “people killed at work accidents”. Partly, lagged publication may also be due to the international transmission process from national to supranational statistical bodies. For example, data on “people killed in accidents at work” are available earlier at the Austrian national level with a similar definition to Eurostat. In March 2021, which was the cut-off-date for the estimation and our nowcasts, the most recent publications at the EU-level by Eurostat (2020) and for Austria by Wegscheider-Pichler and DeCillia (2020a, b) presented the development until the years 2018 and 2019, depending on the indicator. Wegscheider-Pichler and DeCillia (2020a) additionally discussed possible COVID-19 effects on the 17 SDGs using first data for 2020, but without providing values for the SDG indicators. A nowcast of SDG indicators based on the first wave of published quarterly data from the current year, would pull forward an early SDG evaluation by 1 year. Moreover, a nowcast provides expected values for each indicator and therefore also the expected direction of change, i.e. convergence, divergence, or stagnancy with respect to SD objectives. This would offer the opportunity of timely monitoring and—if necessary—the implementation of countervailing measures. In the following example, we will base the nowcasts for the year 2020 on quarterly data until the third or fourth quarter of 2020, a year characterised by the response to the COVID-19 outbreak with substantial swings in economic activity.

The paper is structured as follows: Sect. 2 gives an overview of the annual and quarterly data set and details of the monitoring approach used by Eurostat (2020). Section 3 describes the econometric framework, the results are shown in Sect. 4, and Sect. 5 concludes.

## The EU SDG data set

SDG 8 refers to “Sustained, inclusive and sustainable economic growth, full and productive employment and decent work for all”. The goal is measured with six key indicators and three multipurpose indicators. The multipurpose indicators stem from SDG 1 “End poverty in all its forms everywhere”, SDG 5 “Achieve gender equality and empower all women and girls” and SDG 12 “Ensure sustainable consumption and production patterns”. They cover aspects that are interrelated with SDG 8 and create links between the goals. Eurostat (2020) presents the indicators and evaluates the progress towards SD goals at the EU level. Table 1 lists the indicators collected in SDG 8 and illustrates the short-term trend over the 5-year period 2014–2019 towards or away from the SD objectives for Austria. The middle column of Table 1 presents the compound annual rate of change in the indicators over the reference period. For indicators measured as ratios, Table 1 also reports the annual average change between 2014 and 2019 measured in percentage points.

**Table 1:** Assessment of the indicators' developments in Goal 8

		Sample	2014/2019		
			Compound rate of change	Average change in %-points	Progress towards objective
*Key indicators*					
08_10	Real GDP per capita	1990–2020	+ 1.1		⇧
08_11	Investment share of GDP	1995–2020	+ 1.7	+ 0.4	**⇧**
08_20	Young people neither in employment nor in education and training	2000–2019	–2.2	–0.2	**⇧**
08_30	Employment rate^a^	1995–2020	+ 0.7	+ 0.5	⇧
08_40	Long-term unemployment rate	2002–2019	–6.0	–0.1	**⇧**
08_60	People killed in accidents at work^b^	2010–2018	–3.5	–0.1	**⇧**
*Multipurpose indicators: supplementary indicators of other goals which complement the monitoring of this goal*					
01_41	In work at-risk-of-poverty rate	2003–2019	+ 1.1	+ 0.1	**⇩**
05_40	Inactive population due to caring responsibilities	1995–2019	–1.7	–0.3	**⇧**
12_20	Resource productivity (output per DMC)	1995–2019	+ 0.4		**⇗**

*Source*: Eurostat (2020) and own computation with cut-off date 17 March 2021

^a^Series with national 2020-target

^b^Short-term trend according to published data from 2013 to 2018

The assessment by Eurostat (2020) considers the underlying direction (towards or away from the SD objective) and the pace of the development of the indicator series. Indicators, for which a quantitative EU policy target exits, are assessed with respect to a theoretical path necessary to reach the goal. Eurostat uses arrows as communication tool for the assessment. Their direction does not necessarily indicate the direction of the change of the underlying series, rather it implies progress towards the SD objective by pointing upwards. For example, an increase in “GDP per capita” as well as a decline in “long-term unemployment” are described by upward pointing arrows.

In order to orient the arrows Eurostat (2020, page 356 ff) defines the following thresholds for the compound rate of change over the past 5 years: for indicators without an explicit quantitative target, a change of 1 percent or more in the desired direction indicates a significant progress and is represented by an upward arrow. A stagnation or a change below 1 percent in the desired direction is evaluated as a moderate progress towards SD objectives and represented by an ascending arrow. In contrast, changes in the other direction indicate a movement away from the SD objectives, with a change of less than 1% interpreted as moderate (descending arrow). A decrease by more than 1 percent is a significant movement away from SD objectives, represented by a downward pointing arrow. For indicator series where quantitative EU policy targets exist (like the national 2020-target for the employment rate), the recent compound rate of change over the past 5 years is set in relation to the rate theoretically required to meet the target in time. If the compound rate of change is 95 percent or more of the theoretically required rate, the series indicates a significant progress towards target (upward arrow). In the case this ratio is between 60 and 95%, the indicators development is characterised by moderate progress (ascending arrow). A ratio lying between 0 and 60% indicates a moderate movement away from the target (descending arrow). If the ratio is negative, this implies that the recent short-term trend moves significantly away from target (downward arrow).

The aim of nowcasts is to use data published throughout the current year for an early evaluation of progress towards SDGs during that year. Given the publication calendar of statistical agencies, the earliest possible starting month for a nowcast exercise would be May, making possible a first evaluation of progress towards objectives around June of the current year. In comparison to the publication of the final evaluations by Eurostat and Statistics Austria, this time schedule could pull forward a first discussion about achieving SDGs by 1 year.

As an example, we will provide the results for a nowcasting of indicators in the year 2020 in Sect. 4. These nowcasts are based on a comprehensive data set of quarterly variables available up the third or fourth quarter of 2020. Table 6 in the appendix lists all variables, their sources, and their sample size. Monthly data are aggregated to quarterly frequency. Table 6 also shows which quarterly variables have been used in the dynamic factor models chosen to nowcast SDG 8 indicators. For quarterly variables used in more than one model, the variables are sorted alphabetically within the group. Before using SDG indicators and quarterly variables in the nowcasting systems all data have been transformed. For quarterly series we either used seasonally adjusted versions provided by Statistics Austria or we removed seasonality by applying the Tramo/Seats procedure based on Gomez and Maravall (1996). After performing Adjusted Dickey-Fuller (ADF) tests, all non-stationary series were transformed either into growth rates or—for ratios—into first differences. Then, the series were standardised to mean zero variables with a standard deviation of one.

The economic indicators belonging to SDG 8, such as “real GDP per capita” (SDG 08_10), the “investment share of GDP” (SDG 08_11), and the “employment rate” (SDG 08_30), are subject to regular for- and nowcasts and therefore not addressed in the modelling framework here. Moreover, they are released timely and, given our cut-off date for the estimation, preliminary figures have already been released for 2020. For the other six indicators of SDG 8 we conduct nowcasts to provide a first impression of sustainable development in Austria in the light of the COVID-19 pandemic.

## The model framework

### Dynamic factor models

The use of mixed frequency models is now a standard tool in the short-term analysis of economic fluctuations because information about important indicators is often published with a delay and statistical methods have been developed to handle variables and related indicators published at higher frequencies. Statistical authorities, for example, publish monthly or quarterly data for some of the SDG 8 indicators and thus provide recent data for an early prediction of their expected annual values. Such predictions imply that the target variable must be estimated for the very near future but also its current value and the most recent past in advance of later revisions by statistical authorities. Angelini et al. (2011) describe the nowcasting approaches for the euro area GDP used by the European Central Bank, and Glocker and Kaniovski (2020) show how several nowcasts can be combined into a cluster model for the prediction of GDP in combination with its most important demand and production side components.

We apply two variations of dynamic factor models for our nowcasting of SDG 8 indicators. Either we use a broad set of quarterly variables and apply the Doz et al. (2011) two-step estimator to the data or we concentrate the set of variables to the most significant explanatory variables and apply the Mariano and Murasawa (2003) extension of the Stock and Watson (1991) approach to compute coincident indicators, which can be used for the prediction of the target variable. The general idea behind dynamic factor models is to represent the variation in an observed low-frequency variable, e.g. GDP, with one or more latent variables, capturing the main cross-sectional co-movements in a possibly large panel of quarterly variables.

### Dynamic factor model using a narrow subset of quarterly variables

The first approach follows the work by Stock and Watson (1991) in its extension by Mariano and Murasawa (2003). The following state-space representation of a dynamic factor model

1 $$X_{t} = \Lambda ^{\prime}\Omega \left( L \right)f_{t} + \Theta \left( L \right){E}_{t} ,$$

2 $$\phi \left( L \right)f_{t} = u_{t} ,$$

is estimated by maximum likelihood using the Kalman filter. The mixed frequency structure of the model is solved by transforming the annual SDG indicator into a quarterly time series involving missing observations. The Kalman filter can cope with missing observations, this property also allows for ragged edges in the panel of quarterly variables, i.e. different starting and end dates of the time series resulting from the publication cycle of statistical agencies. The ($\left( {n + 1} \right) \times 1)$ vector $X_{t}$ includes all $n$ observed quarterly time series at time $t$ and the observed low-frequency SDG indicator. The vector $X_{t}$ is a stationary process. $f_{t}$ is a scalar following a stationary process representing the single unobserved common factor and ${E}_{t}$ is a $\left( {\left( {n + 1} \right) \times 1} \right)$ vector stationary process of idiosyncratic measurement errors. ${\Lambda }$ is the $\left( {\left( {n + 1} \right) \times 1} \right)$ matrix of factor loadings and ${\Theta }\left( L \right)$ is a $\left( {\left( {n + 1} \right) \times \left( {n + 1} \right)} \right)$ matrix polynomial in the lag operator capturing dynamics in the measurement error. Let the target variable be the $\left( {n + 1} \right)$th element of $X_{t}$, i.e. the last row, then the element $\lambda_{n + 1}$ in vector $\Lambda$ represents the loading of the unobserved factor on the SDG 8 indicator. We restrict the moving average structure in measurement Eq. (1) to the equation for the SDG indicator, i.e. the matrix ${\Theta }\left( L \right)$ is an identity matrix except for the last element in the $\left( {n + 1} \right)$th row and column, which contains a polynomial in the lag operator.

Given this ordering of variables in vector $X_{t}$, the vector polynomial in the lag operator ${\Omega }\left( L \right)$ has the following shape: the first $n$ elements are ones, such that the common factor is not lagged for all quarterly variables. The last row, on the other hand, reflects the fact that the quarterly variables and the SDG target variable are observed at different frequencies: annually and quarterly. In a quarterly setting the SDG indicator is observed every fourth period. In order to combine the different frequencies in the measurement equation, we follow Mariano and Murasawa (2003) and adapt their frequency conversion (which is between quarters and months) to be applied between years and quarters. Here we assume that the annual data $Y_{t}^{y}$ can be approximated with the geometric mean of the quarterly data $Y_{t}^{q}$:

$$Y_{t}^{y} = \frac{1}{4}(ln Y_{t}^{q} + ln Y_{t - 1}^{q} + ln Y_{t - 2}^{q} + ln Y_{t - 3}^{q} ).$$

Taking the annual (four quarter) difference yields

$$y_{t}^{yoy} = \frac{1}{4}(ln Y_{t}^{q} - ln Y_{t - 4}^{q} ) + \frac{1}{4}(ln Y_{t - 1}^{q} - ln Y_{t - 5}^{q} ) + \frac{1}{4}(ln Y_{t - 2}^{q} - ln Y_{t - 6}^{q} ) + \frac{1}{4}(ln Y_{t - 3}^{q} - ln Y_{t - 7}^{q} )$$

with $ln Y_{t}^{q} - ln Y_{t - 1}^{q} = y_{t}^{qoq}$ the equation can be rewritten as:

3 $$y_{t}^{yoy} = \omega \left( L \right)y_{t}^{qoq} = \frac{1}{4}y_{t}^{qoq} + \frac{2}{4}y_{t - 1}^{qoq} + \frac{3}{4}y_{t - 2}^{qoq} + y_{t - 3}^{qoq} + \frac{3}{4}y_{t - 4}^{qoq} + \frac{2}{4}y_{t - 5}^{qoq} + \frac{1}{4}y_{t - 6}^{qoq} .$$

Applying this frequency conversion, the annual year-on-year growth rate of the SDG indicator series, $y_{t}^{yoy}$, is interpolated from quarter-on-quarter growth rates. It allows us to interpret the development of the interpolated SDG indicator as a year-on-year growth rate as well. This conversion is not only applicable to growth rates but also to first differences of ratios, e.g. the long-term unemployment rate. The conversion formula limits the number of common factors in this model class to $r = 1$.

Finally, the transition Eq. (2) shows the vector autoregressive structure of the common factor with the polynomial in the lag operator $\phi \left( L \right)$ showing the dynamics of the system, and the scalar $u_{t}$ is a structural innovation following a normally independently distributed process with mean zero and constant variance (Mariano and Murasawa 2003). Furthermore, the structural innovations $u_{t}$ are assumed to be uncorrelated with the measurement errors ${E}_{t}$ at all leads and lags.

The system of Eqs. (1) and (2) can be estimated directly by using the Kalman filter to interpolate the missing elements of the SDG indicator and to estimate all parameters of the system at once. Maximum likelihood estimation also allows for parameter tests and a search for a parsimonious model. In our application, we assume that the common factor,$f_{t}$, follows an autoregressive process of at most order $p = 2$, and the number of lags in the polynomial for the measurement error of the SDG indicator, $\varepsilon_{n + 1,t}$, is at most $q = 2$. Even if we keep the order of the model small, the iterations are still very time-consuming. Therefore, we restrict the number of quarterly variables, $n$, to about 15. The Kalman smoother delivers predictions of the missing observations for the SDG indicator and the latent common factor including their near-term forecasts for the remaining quarters of the year. After de-standardizing and re-transforming the SDG indicator, we obtain the nowcasts at the annual level. In the following presentation we will use the acronym *SW-model* for models with a narrow subset of variables and maximum likelihood estimation.

### Dynamic factor model using a broad set of quarterly variables

The dynamic factor model suggested by Mariano and Murasawa (2003) puts computational constraints on the number of quarterly variables, and the frequency conversion limits the number of common factors to $r = 1$. Often many quarterly series are available for one SDG indicator, perhaps featuring more than one common factor. Doz et al. (2011) suggest an alternative two-step estimator for the simplified state space model:

4 $$X_{t} = \Lambda F_{t} + {E}_{t} ,$$

5 $${\Phi }\left( L \right)F_{t} = U_{t} ,$$

allowing for a large number of quarterly time series in $X_{t}$. The evolution of each series is again disaggregated into two orthogonal components: a small number $r$ of common factors, represented as the ($r \times 1$) vector $F_{t}$, capture the main cross-sectional co-movements in the quarterly variables, while the measurement errors are driven by $n + 1$ shocks being specific or local to the series. The relation between common factors and the observed variables in the measurement Eq. (4) is given by the ($\left( {n + 1} \right) \times r$) matrix of loadings $\Lambda$. Given the large potential cross-sectional dimension of the panel, the model is identified, provided the number of common factors, $r$, is small with respect to $n$, and the idiosyncratic components in the $\left( {r \times 1} \right)$ vector of errors $U_{t}$ are only weakly correlated.

The two-step estimator by Doz et al. (2011) first estimates the parameters of the model by simple least squares using the common factors from a principle components analysis as if they were the true common factors in model (4) to (5). In the second step, the parameters are used in a Kalman filter to estimate the parameters in the vector autoregression in the transition equation, the loading of the factors onto the SDG indicator, $\lambda_{n + 1,j}$ for $j = 1,2, \ldots r$, and the variances of the model. The principle components analysis, however, requires a balanced data set, i.e. the starting and end dates of the panel must be identical for all quarterly variables. Therefore, the model is set up in state space form and estimated by the Kalman filter using the factor loadings from the principle component analysis for the balanced panel as if they were known. Under these assumptions, the Kalman smoother can consistently estimate the common factors, $F_{t}$, including their near-term forecasts for the remaining quarters of the year. After de-standardizing and re-transforming the SDG indicator, we obtain the nowcasts at the annual level. This approach is robust against misspecification, if the loadings are estimated by principle components analysis.

The advantages of the Doz et al. (2011) estimator are a larger information set, more flexibility in terms of the number of common factors, and easy and fast computation. In the following, we concentrate on models with at most $r$ = 2 common factors and $p$ = 2 autoregressive lags in the transition equation, using the Eviews subroutine developed in Solberger and Spanberg (2017) for estimation. We will use the acronym *DGR-model* for models with a broad set of quarterly variables in combination with the two-step estimator.

### Model selection

The decision between both model types, the choice of the specification with autoregressive and moving average terms, and the selection of variables to be included in the model is complicated by a partially short sample size for SDG indicators as well as the related quarterly variables. While national accounts data have been compiled within the ESA 2010 since 1995^1^ (cf. Table 1), the annual series for the number of “people killed in accidents at work” starts in 2010. Similarly, while a few quarterly variables start in the first quarter of 1990 (cf. Table 5 in the appendix), the publication of most labour market variables by the Austrian Labour Market Service and Statistics Austria starts in the first quarter of 2004 and some variables from the Federation of Austrian Social Security Institutions begin in 2015. Thus, our model selection search is restricted to a small out of sample period (3–6 years, depending on the length of the underlying series), such that enough observations are left for the estimation of the model.

We start the model selection process with a search for quarterly variables possibly related to an SDG indicator and use a broad variable set to estimate four possible specifications (with respect to the number of factors $r = 1, 2$ and the number of autoregressive lags in the transition equation $p = 1, 2$) of the DGR-model. From these models we collect all one-step ahead forecasts based on pseudo real-time data for the years 2014 through 2019, i.e. we compute the nowcast for the year 2014 based on pseudo real time quarterly information potentially available in spring 2014. The nowcasts can then be used to compute one-step ahead forecast errors for the four possible specifications of the DGR-model. Similarly, we run this exercise with the SW-model. This model type cannot cope with a large set of quarterly data; therefore, we reduce the panel of quarterly variables by estimating a series of bivariate SW-models for all pairs of quarterly variables. We then collect up to 15 quarterly variables producing simultaneously a significant loading onto themselves and onto the SDG indicator and include these variables into a large SW-model. Subsequently, we use a general to specific model search and eliminate insignificant variables from the model, and finally we apply the Kalman smoother to the refined SW-model to compute nowcasts based on the same pseudo real-time data set. These nowcasts are again used to compute one-step ahead forecast errors for six possible specifications of the SW-model ($r = 1$; $p = 1, 2$; $q = 0, 1, 2$).

Given all possible specifications, we compare the root mean squared error (RMSE) for each specification for the period 2014 through 2019^2^ and choose the final nowcasting model as the one with the lowest RMSE among the alternatives (cf. column labelled *DFM* in Table 2). Concentrating the model selection procedure on the out-of-sample predictive performance rather than the in-sample fit, leads to DFMs better suited to produce a high nowcasting accuracy. Moreover, Table 2 also presents the RMSE based on simple random walk forecasts of SDG 8 indicators and the RMSE from auto-selected ARIMA based one-step-ahead forecasts estimated with annual data. The comparison with the RMSE from the DFM model shows that available quarterly information in spring improves the forecasting performance, except for the indicator for workplace security, i.e. the number of “people killed in accidents at work”. For this series the sample size for the estimation is small, impairing the nowcasting performance in comparison to simpler univariate models. For the other series, where the RMSEs of the DFMs are smaller compared to each competing model, we conduct Diebold and Mariano (1995) tests in the modified version by Harvey et al. (1997). At conventional significance levels, the nowcast precision of the DFM models is not significantly different from the competing models. The small number of nowcasts (between three and six), however, reduces the power of these tests and we additionally provide some p-values in Table 2. Details and the preferred DFM model structure for each SDG indicator are given in Table 2.

**Table 2:** Model specifications for each SDG indicator series

		Modell	Number of			Correct signs	Root mean squared error (RMSE)		
			Factors	Autoregressive	Moving average		DFM	ARIMA	Random Walk
*Key indicators*									
08_10	Real GDP per capita	–							
08_11	Investment share of GDP	–							
08_20	Young people neither in employment nor in education and training (in %)	SW	1	2	0	3 out of 6	0.43	0.47	0.44
08_30	Employment rate	–							
08_40	Long-term unemployment rate (in %)	SW	1	1	1	3 out of 6	0.23	0.25	0.25
08_60	People killed in accidents at work (in %)	DGR	1	1	0	3 out of 6	0.36	0.34	0.34
*Multipurpose indicators*									
01_41	In work at-risk-of-poverty rate (in %)	DGR	2	1	0	2 out of 4	0.54	0.55	0.54
05_40	Inactive population due to caring responsibilities (in %)	DGR	2	2	0	3 out of 3	0.42	0.47	0.69
12_20	Domestic material consumption (DMC in thousand tons	DGR	2	2	0	5 out of 6	4189	6599	4357

The estimation samples vary according to data availability, cf. Tables 1, 6. DGR refers to models using a broad set of high-frequency indicators and the two-step estimator suggested by Doz et al. (2011), SW refers to models using a narrow set of high-frequency indicators and the maximum likelihood estimator by Stock and Watson (1991) in the extension of Mariano and Murasawa (2003). Mariano-Diebold tests in the modified version by Harvey et al. (1997) show that the differences in nowcast precision between DFM and alternative models are not significant at conventional significance levels. Given the low power of these tests, resulting from the small number of nowcast errors (between 3 and 6), we also present the lowest *p* values. The *p* values are 0.21 (DFM for 12_20 against ARIMA); 0.32 (DFM for 05_40 against random walk); and 0.40 (DFM for 08_40 against random walk); the remaining *p* values are above 0.50

The evaluation of SDGs often refers to the convergence towards a target level of the specific SDG indicator or a development of the indicator signaling better living conditions. Therefore, the quality of forecasting models for SDG indicators can also be evaluated according to their ability to nowcast the correct direction of change. We present the number of correct out of sample directional changes for the respective model with the lowest RMSE in the middle column of Table 2. Although the number of correct directional changes is quite high for some of the variables, conclusions should be drawn with caution because the sample size for our out of sample forecasts is extremely small and consequently a single large forecast error may dominate the model selection procedure. On the other hand, given the few out of sample realisations available, we hesitate to directly reject a model with low RMSE if the number of correctly predicted signs is low.

## Results

### Estimation results

The nowcasting system we propose, relates quarterly variables published throughout the year to each of the six SDG indicators in a separate dynamic factor model. Table 3 highlights the eight most important quarterly series for each model, sorted according to the highest positive and negative loading on the SDG indicator. Among them are detailed labour market and income data, national accounts data, industrial production, net exports of raw material, the number of care allowance recipients according to different classifications and demographic data. The full set of quarterly variables together with their source and available sample size is reported in Table 6 in the appendix.

**Table 3:** Quarterly-frequency variables with highest factor loadings for each SDG indicator

		No. of series used	Selection of quarterly series according to highest coefficient estimate	
			Positive loading	Negative loading
*Key indicators*				
08_10	Real GDP per capita	–	Preliminary values for 2020 already released	
08_11	Investment share of GDP	–	Preliminary values for 2020 already released	
08_20	Young people neither in employment nor in education and training	5	Unemployment rate for persons aged 15–24, in percent of labour force of the same age/unemployment rate for persons with non-Austrian citizenship aged 15–24, in percent of labour force with same characteristics/unemployment rate for persons with non-Austrian country of birth aged 15–24, in percent of labour force with same characteristics/unemployment rate for persons aged 15–24, in percent of dependent labour force/young persons aged 15–29 not in employment, education or training (NEET), percent of population of the same age	
08_30	Employment rate	–	Preliminary values for 2020 already released	
08_40	Long-term unemployment rate	5	Long-term unemployed persons, percent of labour force	Business survey, current business situation construction sector, balance/business survey, current business situation manufacturing sector, balance/industrial labour input, working hours manufacturing sector, 2015 = 100/business survey, production constraint lack of workforce construction sector, percent
08_60	People killed in accidents at work	72	Employment rate of men aged 20–64, percent of population of the same age	Long-term unemployed women, percent of labour force/long-term unemployed persons, percent of labour force/unemployment rate for persons with only compulsory school leaving certificate, in percent of dependent labour force/long-term unemployed men, percent of labour force/young persons aged 15–29 not in employment, education or training (NEET), persons/unemployed persons aged 15–24/unemployed persons with only compulsory school leaving certificate
*Multipurpose indicators*				
01_41	In work at-risk-of-poverty rate	62	Employed persons from non-EU member states	Unemployed persons aged 15–24/unemployment rate for persons aged 15–24, in percent of dependent labour force/unemployment rate for persons with only compulsory school leaving certificate, in percent of dependent labour force/long-term unemployed persons, all status/unemployed persons aged 15–24/unemployed women aged 15–24, persons/long-term unemployed persons
05_40	Inactive population due to caring responsibilities	11	Child benefit recipients with valid employment contract, women / Population aged 6–14 at begin of quarter/part-time employed persons aged 20–64, in percent of total employment	Population aged 0–5 at begin of quarter/salaried employees and civil servants aged 15–64, men, in percent of population of the same age group/salaried employees and civil servants aged 15–64, women, in percent of population of the same age group/workers aged 15–64, men, in percent of population of the same age group/workers aged 15–64, women, in percent of population of the same age group
12_20	Domestic material consumption (DMC)	11	Net exports of crude materials and fuels, Euro/Net exports of mineral crude materials, Euro/industrial production—manufacture of products of wood, cork, straw and plaiting materials, 2015 = 100/net exports of textile crude materials, Euro/wood harvest/market consumption of fuels (diesel, regular gasoline, premium gasoline)/net exports of solid fuels, Euro	

Table 4 reports the parameter estimates of the factor loadings on the SDG indicator, i.e. the coefficients for ${\lambda }_{n+1,j}$ in Eqs. (2) and (5), respectively, together with their p-values. In three of the six dynamic factor models one common factor is extracted, leaving us with a scalar ${f}_{t}$ and a single element ${\lambda }_{n+1}$. Three of the models feature two common factors, consequently ${F}_{t}$ has two columns and $j=1, 2$. In the case of the nowcasting model for the “Young people neither in employment nor in education and training” (SDG 08_20), the coefficient of the factor is positive and significant. Table 4 also shows that even comparatively high estimates for factor loadings on SDG indicators feature high *p* values. Small sample sizes (cf. Table 1) are one possible reason for this result. Possibly high correlation between quarterly series is another explanation. While high correlation is an advantage for the estimation of a common factor, the estimation procedure for SW-models uses the Kalman filter to estimate the unobserved factor, its loadings and the variances simultaneously. In case of high multicollinearity, this leads to high variance estimates for the loadings. The comparison of RMSEs for nowcasts based on the preferred dynamic factor models with simpler alternatives like random walks or ARIMA based one-step-ahead forecasts, nevertheless, reveals a better forecasting performance for all but one SDG indicator, even this is not significant at conventional levels. The value of short-term information results either directly from the factor loadings documented in Table 4 or from the moving average terms in the measurement Eq. (1).

**Table 4:** Dynamic factor models, estimation results

		F1		F2	
		Coefficient	*p* value	Coefficient	*p* value
*Key indicators*					
08_10	Real GDP per capita	–	–	–	–
08_11	Investment share of GDP	–	–	–	–
08_20	Young people neither in employment nor in education and training	0.15	0.02	–	–
08_30	Employment rate	–	–	–	–
08_40	Long-term unemployment rate	0.01	0.80	–	–
08_60	People killed in accidents at work	0.18	0.73	–	–
*Multipurpose indicators*					
01_41	In work at-risk-of-poverty rate	– 0.23	0.86	– 0.08	0.82
05_40	Inactive population due to caring responsibilities	– 0.29	0.55	– 0.06	0.66
12_20	Domestic material consumption (DMC)	0.73	0.23	0.03	0.96

The estimation samples vary according to data availability, cf. Table 1. For a complete specification of the dynamic factor model compare Table 2

### Early monitoring of SDGs in the light of the COVID-19 pandemic

Our assessment of SDG indicators closely follows the framework designed by Eurostat (2020) for the presentation of the EU Sustainable Development Goals but is confined to Austria. In order to discuss the implications of the COVID-19 crisis for SDG 8 we extent the short-term trend for SDG 8 indicators in Austria by adding the nowcast for 2020 to the 5-year period shown in the survey table for SDG 8 in Eurostat (2020). We indicate the expected change over the past 6-year trajectory by arrows representing improvement, stagnation, or deterioration (Table 5), and compare the extended short-term trends from our nowcasting example with those relying only on the published data until 2019 (Table 1).

**Table 5:** Assessment of the indicators' developments in Goal 8 including Nowcasts

		2014/2020		
		Compound rate of change	Average change in %-points	Progress towards objective
*Key indicators*				
08_10	Real GDP per capita	– 0.3		⇘
08_11	Investment share of GDP	+ 1.8	+ 0.4	⇧
08_20	Young people neither in employment nor in education and training	– 1.1	– 0.1	⇧
08_30	Employment rate^a^	+ 0.3	+ 0.2	⇗
08_40	Long-term unemployment rate	– 4.8	– 0.1	⇧
08_60	People killed in accidents at work^b^	– 6.6	– 0.2	⇧
*Multipurpose indicators*				
01_41	In work at-risk-of-poverty rate	+ 3.5	+ 0.3	⇩
05_40	Inactive population due to caring responsibilities	– 2.4	– 0.5	⇧
12_20	Resource productivity (output per DMC)	– 0.4		⇘

*Source*: Eurostat (2020) and own computation with cut-off date 17 March 2021

^a^Series with national 2020-target

^b^Short-term trend according to published data from 2013 to 2018. The extension by our nowcasts covers the period 2013–2020

The COVID-19 pandemic and the containment measures led to a massive decline in economic output in 2020. The period of continuous growth in “per capita GDP” (SDG 08_10) observed between 2014 and 2019 (compound rate of change of 1.1%) ended abruptly, and the assessment in 2020 turned from significant progress towards objective towards moderate divergence from SD objective (average short-term trend of -0.3%). Also, investment demand shrank in 2020, but to a lesser extent than GDP as the economic crisis was driven to a large extend by the decline in private consumption. Consequently, the “investment share of GDP” (SDG 08_11) further increased in 2020. The investment share follows an upward trend since 2010 with an average increase of 0.4 percentage points per year since 2014. Investment activity improves economic growth opportunities both in the short and the medium to long-term future by enhancing productive capacity. The upward trend is positive and recorded as significant progress towards SD objectives.

**Fig. 1:**
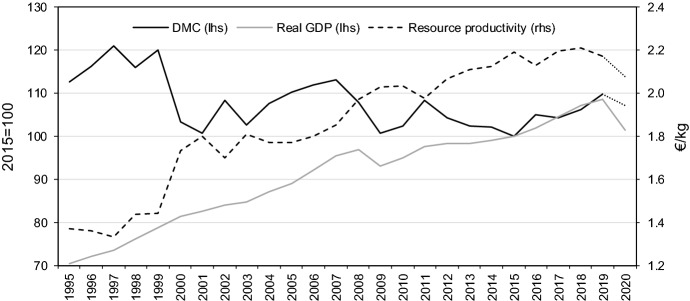
Evolution of resource productivity and domestic material consumption (DMC) *Source*: Eurostat, Statistics Austria. The dotted sections represent the nowcast results for 2020.

Economic growth in Austria has become more sustainable in the past, with natural resources used more efficiently. Between 2014 and 2019 “resource productivity” (SDG 12_20) increased on average by 0.4% (cf. Table 1). The indicator is measured as output per domestic material consumption, where the latter refers to the total amount of materials used in the economy. It is defined as the quantity of raw materials taken from the domestic territory of the economy adjusted by adding all physical imports minus all physical exports. To estimate the dynamic factor model for domestic material consumption, quarterly data on net exports of various crude materials, fuels as well as production data are used (cf. Tables 3, 6). There is some empirical evidence of a positive correlation between economic growth and domestic material consumption (e.g. Agnolucci et al. 2017). A more efficient use of resources implies a weakening or reversal of this relationship. This is also part of the European Green Deal, a new growth strategy that aims to transform the EU into a fair and prosperous society, with a modern, resource-efficient and competitive economy where economic growth is decoupled from resource use (European Commission 2019). Periods of decoupling of domestic material consumption from GDP (either absolute in the sense of GDP growth coinciding with declining material consumption, or relative with domestic material consumption increasing less than GDP) were observed in Austria between 2012/2015 and 2017/2018. During the recession in 2020 domestic material consumption fell, but not as sharp as GDP (Fig. 1). This is observed both, on the base of our nowcast, and on the base of first published data by Eurostat in summer 2021. The COVID-19 pandemic and the measures to contain it, dramatically weighed on expenditures for services (like food service and accommodation as well as body related services, arts and entertainment). They are not as material intensive as demand for investment and consumption goods. Thus, the relation between GDP and domestic material consumption deteriorated in 2020. Between 2014 and 2020 “resource productivity” declined on average by 0.4%. In the assessment this is indicated by a movement away from the SD objective.

The labour market responded extremely fast and negatively to the COVID-19 crisis. Labour market data showed a strong increase in unemployment registrations starting already by mid-March, i.e. immediately after the implementation of containment measures (cf. press release by the Austrian Labour Market Service from 1 April 2020). Final data for the “employment rate” (SDG 08_30) show a strong decline by 1.3 percentage points in 2020, ending a steady upward development since 2014. The 2020 outcome reduced the average annual change from+ 0.5 percentage points (cf. Table 1) over the period 2014 through 2019 to+ 0.2 percentage points (2014–2020, cf. Table 5). The Europe 2020 target for Austria of 77% was missed. At the same time, short-term data for “young people neither in employment nor in education and training” (SDG 08_20, NEETs) and the “long-term unemployment rate” (SDG 08_40) started to increase in the second quarter of 2020. Both quarterly variables are included in the respective model (cf. Table 3) to estimate the annual SDG indicator series. The NEETs belong to a hard-to-place group in the labour market and their integration worked well until 2019 (cf. Table 1). Our nowcast for 2020 signals an increase by 0.4 percentage points towards 8.7%, which was surpassed by the realised value for 2020 of 9.5%. With respect to the long-term unemployed our nowcast indicated an increase as well, which was also outpaced by the final value of 1.3%. Even though the nowcasts point towards a worsening of the conditions for vulnerable groups among the unemployed, the arrows in Table 5 remain unaffected and still show continued progress toward the SD objectives.

Similarly, the indicator “inactive population due to caring responsibilities” (SDG 05_40) is assessed to develop favourable in the short run. The rate is on a downward trend since 2007, where it recorded 27.4%. The decline continues in 2020, where the nowcasted rate (17.3%) closely matches finally published data (17.2%). Taking a closer look to disaggregated data, actual published data show an ongoing decline for women, while the male inactive population due to caring responsibilities increased in 2020 when the COVID-19 pandemic had hit the labour market.

To observe the aspect of decent work, SDG 8 is complemented by the indicator “in work at-risk-of poverty rate” (SDG 01_41). Since 2014 no improvement has been observed here and the nowcast for 2020 shows a dramatic increase from 7.6% towards 8.8%. Actually, the realised value for 2020 was 7.2%, signaling an improvement of poverty at work during the COVID-19 crisis. This counterintuitive outcome is a result of the definition of the indicator as the ratio of persons earning less than the 60% of the median income to total employment. The sharp reduction in overall employment was not evenly distributed across income deciles. Whereas well-paid jobs were often supported by short-time work or they were not at all affected by closure rules and mobility restrictions due to the wide-spread use of homeworking, low-paid jobs have been terminated. Consequently, the number of low-paid employees declined even more sharply than total employment, resulting in a lower ratio. The nowcasting model, in contrast, interprets deteriorating labour market conditions, as reflected in both factors, as a move towards more income inequality among wage earners. The second indicator covering aspects of decent work refers to “people killed in accidents at work” (SDG 08_60) where strong improvements were recorded since 2014 (cf. Table 1). The nowcast for 2020 uses more recent information from national annual data for 2019 and adds the information contained in a broad set of quarterly labour market data and business cycle indicators. Lower economic activity consequently leads to a reduction in injuries and reinforces the previous trend towards improving working conditions. Our nowcast mainly picks up business activity, while political interventions such as the introduction of further protective measures at the workplace are hardly possible to include in this kind of framework.

The effects of the COVID-19 pandemic and the economic crises are clearly visible in the nowcasts of most indicators for 2020. Extending the evaluation period to 2020, either leads to a worsening assessment for the development of SDG 8 indicators or leaves the assessment constant.

## Conclusions

The publication of indicators for Sustainable Development Goals in Europe is a recent phenomenon and consequently many time series used to evaluate the development towards SDGs are short and published with a considerable delay. Although, even delayed publication represents an improvement against the previous situation, we suggest complementing conventional short-term forecasts of economic activity with nowcasts for those indicators not covered in conventional forecasts. This makes it possible to pull forward a first evaluation of sustainable development and progress towards the mid of the current year, allowing for a swift policy response in case of a severe target violation.

The set-up of a nowcasting system for annual SDG 8 indicators is not as straightforward as in the case of quarterly GDP, because the time series for annual indicators and many of the explanatory quarterly series used in the dynamic factor models are short. This prevents a thorough out of sample evaluation of the nowcast accuracy, and the model selection process entails more uncertainty than usual. It also produces nowcasting models with ambiguous statistical properties. A first analysis of the out of sample nowcast accuracy with respect to SDG 8 indicators for Austria over the period 2014 through 2019 shows some weaknesses of the models, particularly in terms of coefficient significance tests. If we compare the forecasting performance to simple random walk forecasts and auto-selected ARIMA based forecasts, the RMSEs from the dynamic factor models are smaller in general. The difference, though, turns out not to be significant at conventional levels. Although nowcast-precision tests based on few nowcast errors suffer from low power, simpler nowcasting strategies may still be a viable alternative to dynamic factor models. Further nowcasting rounds will make the difference in the relative nowcast precision clearer. We based our model evaluation on intra-year information available in spring of the relevant year. As the year progresses, more and more intra-year information becomes available and by adding this information the performance of nowcasting models should improve further.

Comparing the nowcasting results for the year 2020 with realised data first published by Eurostat in summer 2021 shows that the predictions from our nowcasting system appear reliable, and we conclude that our results provide a useful input for an early assessment of sustainable development in the light of the COVID-19 pandemic. The nowcasting of Austrian SDG 8 indicators also reveals synergies of using quarterly labour market data in models for indicators assigned to SDG 1 and SDG 5.

After the start of the COVID-19 crisis in March 2020, Austrian decision makers responded very quickly to the expected deterioration of the economy and the negative consequences for vulnerable groups. Among the measures particularly relevant with respect to low income households were the generous short-time work program, the hardship fund (Härtefallfonds), supplementary payments to unemployment benefits, the children bonus, and the Corona family hardship compensation. Due to the sharp downturn in economic activity these measures have been implemented between April (short-time work, hardship fund, and family compensation), June (supplementary benefit), and September (children bonus) 2020, thus mostly at a time when the nowcasts of SDG 8 for 2020 based on quarterly data would have been prepared rather than published. Insofar this kind of nowcasts would not have been helpful during the design stage and the public discussion of countervailing measures in the first half of 2020. In a sense, policy was quicker than statistical analysis would have been. Nevertheless, the introduction of the family hardship compensation could have been justified by increased poverty indicators in our nowcasts, and most of the implemented measures had a terminal date attached. Thus, nowcasts could deliver helpful information for the decision to continue a program. Finally, the transfer payments associated with the aforementioned programs amounted to 7.5 bn € or 2% of GDP until the end of 2020. Payments of this size require public scrutiny and the compact presentation of timely updated economic and social indicators in SDG 8 provides useful information.

Surprisingly, a single year with a severe shock like the COVID-19 crisis does change the short-term assessment of progress towards SDG 8. Adding the nowcasts for 2020 to the time series and extending the span over which we compute compound rates of change by one year towards 2014–2020, turns around many of the arrows signalling progress towards sustainable and inclusive growth, as compared to the evaluation based only on realisations over the period 2014–2019.

Even if SDG 8 has a strong economic focus and therefore several indicators are regularly forecasted or observed at monthly or even weekly frequency, it also includes social and ecological indicators such as “in work at-risk-of-poverty rate” and the “resource productivity”, where information during the year is scarce. This lack of timely information also applies to other SDGs where no institution carries out regular forecasts. In these cases, data are often less frequently available or lagging more behind. The nowcasting approach we suggest here for SDG 8 can also be extended to these SDG indicators to provide policy makers and the interested public with more recent information.

Another conclusion arising from our nowcasting exercise is the critical importance of a stable set of indicators for the quality of nowcasts. On the one hand, from a purely statistical perspective, stable sets of indicators facilitate estimation and testing. Also, in terms of information diffusion, a stable set of indicators allows a continuous evaluation of targets and improves their usefulness as communication tools, particularly, if they become better known and more established in the media. On the other hand, in order to respond to new policy developments and the evolvement of new methodologies and data sources, the European Commission and Eurostat revise or extent the set of indicators, thus creating a lively body of data. The last comprehensive review of the indicator framework took place in early 2020, the next review is announced for 2025 (Eurostat 2020). This approach has the advantage of flexibly adjusting the information needed in the discussion of open issues, but it impairs and distracts public attention by permanently changing and increasing the information set and it makes nowcasting less precise.

## Data Availability

Available on request.

## Appendix

See Table 6.

**Table 6:** List of high-frequency variables

Variable	Source	Sample size	08_20	08_40	08_60	01_41	05_40	12_20
Unemployment rate for persons with non-Austrian country of birth aged 15–24, in percent of labour force with same characteristics	Statistics Austria (LFS)	IQ04 – IIIQ20	1		1	1		
Unemployment rate for persons with non-Austrian citizenship aged 15–24, in percent of labour force with same characteristics	Statistics Austria (LFS)	IQ04 – IIIQ20	1		1	1		
Unemployment rate for persons aged 15–24, in percent of labour force of the same age	Statistics Austria (LFS)	IQ04 – IIIQ20	1		1	1		
Business survey, current business situation—construction sector, balance	WIFO	1M96 – 2M21		1	1			
Business survey, current business situation—manufacturing sector, balance	WIFO	1M96 – 2M21		1	1			
Business survey, production constraint lack of workforce—construction sector, percent	WIFO	1M96 – 2M21		1	1			
Industrial labour input, working hours—manufacturing sector, 2015 = 100	Statistics Austria	1M05 – 12M20		1	1			
Long-term unemployed persons	Austrian Labour Market Service	IQ04 – IVQ20			1	1		
Long-term unemployed persons, all status	Austrian Labour Market Service	IQ07 – IVQ20			1	1		
Long-term unemployed persons, percent of labour force	Austrian Labour Market Service, Federation of Austrian Social Security Institutions	1M03 – 1M21		1	1			
Unemployed men aged 15–24, type of living, persons	Statistics Austria (LFS)	IQ04 – IIIQ20			1	1		
Unemployed persons aged 15–24	Austrian Labour Market Service	IQ04 – IVQ20			1	1		
Unemployed persons aged 15–24	Statistics Austria (LFS)	IQ04 – IIIQ20			1	1		
Unemployed persons for a duration between 6 and 12 months	Austrian Labour Market Service	IQ97 – IVQ20			1	1		
Unemployed persons for a duration between 6 and 12 months	Statistics Austria (LFS)	IQ04 – IIIQ20			1	1		
Unemployed persons for a duration of more than 12 months	Austrian Labour Market Service	IQ97 – IVQ20			1	1		
Unemployed persons for a duration of more than 12 months	Statistics Austria (LFS)	IQ04 – IIIQ20			1	1		
Unemployed persons with less than ISCED-0-2 educational attainment level	Statistics Austria (LFS)	IQ04 – IIIQ20			1	1		
Unemployed persons with only compulsory school leaving certificate	Austrian Labour Market Service	IQ04 – IVQ20			1	1		
Unemployed persons with only compulsory school leaving certificate	Statistics Austria (LFS)	IQ04 – IIIQ20			1	1		
Unemployed women aged 15–24, persons	Statistics Austria (LFS)	IQ04 – IIIQ20			1	1		
Unemployed women aged 15–24, type of living, persons	Statistics Austria (LFS)	IQ04 – IIIQ20			1	1		
Unemployment rate for persons aged 15–24, in percent of dependent labour force	Austrian Labour Market Service	IQ04 – IVQ20	1		1			
Unemployment rate for persons with an unemployment duration between 6 and 12 months, in percent of labour force	Statistics Austria (LFS)	IQ04 – IIIQ20			1	1		
Unemployment rate for persons with an unemployment duration of more than 12 months, in percent of labour force	Statistics Austria (LFS)	IQ04 – IIIQ20			1	1		
Unemployment rate for persons with less than ISCED 0–2 educational attainment level, in percent of labour force with ISCED 0–2	Statistics Austria (LFS)	IQ04 – IIIQ20			1	1		
Unemployment rate for persons with only compulsory school leaving certificate in percent of labour force with same characteristics	Statistics Austria (LFS)	IQ04 – IIIQ20			1	1		
Unemployment rate for persons with only compulsory school leaving certificate, in percent of dependent labour force	Austrian Labour Market Service	IQ04 – IVQ20			1	1		
Young persons aged < 25 supported by the Austrian labour market service, persons	Austrian Labour Market Service	IQ07 – IVQ20			1	1		
Young persons aged 15–29 not in employment, education or training (NEET), persons	Statistics Austria (LFS)	IQ06 – IIIQ20			1	1		
Young persons aged 15–29 not in employment, education or training (NEET), percent of population of the same age	Statistics Austria (LFS)	IQ06 – IIIQ20	1					
Business survey, construction activity in the last 3 months—construction sector, balance	WIFO	5M03 – 2M21			1			
Business survey, current capacity utilisation—manufacturing sector, percent	WIFO	IQ96 – IVQ20			1			
Business survey, current finished goods inventories—manufacturing sector, balance	WIFO	1M96 – 2M21			1			
Business survey, ensured production time—construction sector, months	WIFO	IQ96 – IVQ20			1			
Business survey, ensured production time—manufacturing sector, months	WIFO	IQ96 – IVQ20			1			
Business survey, foreign orders sufficient or more—manufacturing sector, percent	WIFO	1M96 – 2M21			1			
Business survey, individual current business situation—construction sector, balance	WIFO	IQ96 – IVQ20			1			
Business survey, individual current business situation—manufacturing sector, balance	WIFO	IQ96 – IVQ20			1			
Business survey, order book levels in the last 3 months—manufacturing sector, balance	WIFO	IQ96 – IVQ20			1			
Business survey, orders sufficient or more—construction sector, percent	WIFO	1M96 – 2M21			1			
Business survey, orders sufficient or more—manufacturing sector, percent	WIFO	1M96 – 2M21			1			
Business survey, production capacity sufficient or more—manufacturing sector, percent	WIFO	IQ96 – IVQ20			1			
Business survey, production constraint financing problems—construction sector, percent	WIFO	1M96 – 2M21			1			
Business survey, production constraint financing problems—manufacturing sector, percent	WIFO	IIIQ03 – IVQ20			1			
Business survey, production constraint lack of demand—manufacturing sector, percent	WIFO	IQ96 – IVQ20			1			
Business survey, production constraint lack of material/capacity—construction sector, percent	WIFO	5M03 – 2M21			1			
Business survey, production constraint lack of material/capacity—manufacturing sector, percent	WIFO	IQ96 – IVQ20			1			
Business survey, production constraint lack of orders—construction sector, percent	WIFO	1M96 – 2M21			1			
Business survey, production constraint lack of workforce—manufacturing sector, percent	WIFO	IQ96 – IVQ20			1			
Business survey, production constraint weather conditions—construction sector, percent	WIFO	1M96 – 2M21			1			
Business survey, production in the last 3 months—manufacturing sector, balance	WIFO	1M96 – 2M21			1			
Dependent active employment in construction sector, persons	Federation of Austrian Social Security Institutions	1M08 – 1M21			1			
Dependent active employment in labour leasing, persons	Federation of Austrian Social Security Institutions	1M08 – 1M21			1			
Dependent active employment in manufacturing sector, persons	Federation of Austrian Social Security Institutions	1M08 – 1M21			1			
Employment rate of men aged 20–64, percent of population of the same age	Eurostat, Statistics Austria (LFS)	IQ95 – IIIQ20			1			
Employment rate of persons aged 20–64, percent of population of the same age	Eurostat, Statistics Austria (LFS)	IQ95 – IIIQ20			1			
Employment rate of women aged 20–64, percent of population of the same age	Eurostat, Statistics Austria (LFS)	IQ95 – IIIQ20			1			
Industrial labour input, working hours—construction sector, 2015 = 100	Statistics Austria	1M05 – 12M20			1			
Industrial production—construction sector, 2015 = 100	Statistics Austria	1M96 – 12M20			1			
Industrial production—manufacturing sector, 2015 = 100	Statistics Austria	1M96 – 12M20			1			
Industrial turnover—construction sector, 2015 = 100	Statistics Austria	1M05 – 12M20			1			
Industrial turnover—manufacturing sector, 2015 = 100	Statistics Austria	1M05 – 12M20			1			
Long-term unemployed men aged 15–74, percent of active population	Eurostat, Statistics Austria (LFS)	IQ95 – IIIQ20			1			
Long-term unemployed men, percent of labour force	Austrian Labour Market Service, Federation of Austrian Social Security Institutions	1M03 – 1M21			1			
Long-term unemployed persons aged 15–74, percent of active population	Eurostat, Statistics Austria (LFS)	IQ95 – IIIQ20			1			
Long-term unemployed persons, all status	Austrian Labour Market Service	IQ07 – IVQ20			1			
Long-term unemployed women aged 15–74, percent of active population	Eurostat, Statistics Austria (LFS)	IQ95 – IIIQ20			1			
Long-term unemployed women, percent of labour force	Austrian Labour Market Service, Federation of Austrian Social Security Institutions	1M03 – 1M21			1			
Real GDP per capita in Euro	Statistics Austria	IQ96 – IIIQ20			1			
Road traffic accidents, persons fatally injured	Statistics Austria	1M12 – 9M20			1			
Road traffic accidents, persons injured	Statistics Austria	1M12 – 9M20			1			
Road traffic accidents, total number	Statistics Austria	1M12 – 9M20			1			
Daily rate for unemployment benefit in Euro	Austrian Labour Market Service	IQ02 – IIIQ20				1		
Daily rate of emergency unemployment assistance in Euro	Austrian Labour Market Service	IQ02 – IIIQ20				1		
Employed men aged 15–24, type of living, persons	Statistics Austria (LFS)	IQ04 – IIIQ20				1		
Employed men aged 15–64 with educational attainment level ISCED 3–8, persons	Statistics Austria (LFS)	IQ04 – IIIQ20				1		
Employed men aged 20–34, persons	Statistics Austria (LFS)	IQ04 – IIIQ20				1		
Employed persons aged 15–64 with educational attainment level ISCED 3–8	Statistics Austria (LFS)	IQ04 – IIIQ20				1		
Employed persons aged 20–34	Statistics Austria (LFS)	IQ04 – IIIQ20				1		
Employed persons from non-EU member states	Federation of Austrian Social Security Institutions	IQ08 – IVQ20				1		
Employed women aged 15–24, type of living, persons	Statistics Austria (LFS)	IQ04 – IIIQ20				1		
Employed women aged 15–64 with educational attainment level ISCED 3–8, persons	Statistics Austria (LFS)	IQ04 – IIIQ20				1		
Employed women aged 20–34, persons	Statistics Austria (LFS)	IQ04 – IIIQ20				1		
Employed women aged 25–44, persons	Statistics Austria (LFS)	IQ04 – IIIQ20				1		
Participation rate of men aged 15–64 with educational attainment level ISCED 3–8, percent of male population of the same age	Statistics Austria (LFS)	IQ04 – IIIQ20				1		
Participation rate of persons aged 15–64 with educational attainment level ISCED 3–8, percent of population of the same age	Statistics Austria (LFS)	IQ04 – IIIQ20				1		
Participation rate of women aged 15–64 with educational attainment level ISCED 3–8, percent of female population of the same age	Statistics Austria (LFS)	IQ04 – IIIQ20				1		
Persons in minor employment	Federation of Austrian Social Security Institutions	IIIQ95 – IVQ20				1		
Persons in minor employment with freelance contract	Federation of Austrian Social Security Institutions	IVQ02 – IVQ20				1		
Pupils and students aged 15–24, female persons	Statistics Austria (LFS)	IQ04 – IIIQ20				1		
Pupils and students aged 15–24, male persons	Statistics Austria (LFS)	IQ04 – IIIQ20				1		
Recipients of disability pension under accident insurance, persons	Federation of Austrian Social Security Institutions	IQ04 – IVQ20				1		
Recipients of disability pension, persons	Federation of Austrian Social Security Institutions	IQ04 – IVQ20				1		
Recipients of pensions for reduced ability to work with related compensatory allowances, persons	Federation of Austrian Social Security Institutions	IQ04 – IVQ20				1		
Recipients of pensions for reduced ability to work with related derelict allowances, persons	Federation of Austrian Social Security Institutions	IQ04 – IVQ20				1		
Recipients of pensions with related compensatory allowances, persons	Federation of Austrian Social Security Institutions	IQ04 – IVQ20				1		
Recipients of pensions with related derelict allowances, persons	Federation of Austrian Social Security Institutions	IQ04 – IVQ20				1		
Recipients of the needs-based minimum benefit, persons	Austrian Labour Market Service	IQ12 – IVQ20				1		
Share of population aged 30–34 with degree from a university/university of applied science, percent of population of the same age	Statistics Austria (LFS)	IQ04 – IIIQ20				1		
Share of population aged 30–34 with educational attainment level ISCED 5–8, percent of population of the same age	Statistics Austria (LFS)	IQ04 – IIIQ20				1		
Share of population with degree from a secondary school/university/university of applied science, percent of population	Statistics Austria (LFS)	IQ04 – IIIQ20				1		
Share of population with degree from a university/university of applied science, percent of population	Statistics Austria (LFS)	IQ04 – IIIQ20				1		
Share of population with educational attainment level ISCED 5–8, percent of population	Statistics Austria (LFS)	IQ04 – IIIQ20				1		
Sick leaves, salaried employees, persons	Federation of Austrian Social Security Institutions	IQ04 – IVQ20				1		
Sick leaves, workers, persons	Federation of Austrian Social Security Institutions	IQ04 – IVQ20				1		
Unemployed men aged 15–24, persons	Statistics Austria (LFS)	IQ04 – IIIQ20				1		
Unemployed persons aged 15–24 with non-Austrian citizenship	Statistics Austria (LFS)	IQ04 – IIIQ20				1		
Unemployed persons aged 15–24 with non-Austrian country of birth	Statistics Austria (LFS)	IQ04 – IIIQ20				1		
Unemployed women seeking part-time work only, persons	Statistics Austria (LFS)	IQ04 – IIIQ20				1		
Unemployment rate for persons aged 15–24, in percent of dependent labour force	Austrian Labour Market Service	IQ04 – IVQ20				1		
Care allowance recipients, level 3, persons	Federation of Austrian Social Security Institutions, Statistics Austria	1M15 – 1M21					1	
Care allowance recipients, level 4, persons	Federation of Austrian Social Security Institutions, Statistics Austria	1M15 – 1M21					1	
Child benefit recipients with valid employment contract, men	Austrian Federal Chancellery	4M90 – 1M21					1	
Child benefit recipients with valid employment contract, women	Austrian Federal Chancellery	1M90 – 1M21					1	
Part-time employed persons aged 20–64, in percent of total employment	Eurostat	IQ95 – IIIQ20					1	
Population aged 0–5 at begin of quarter	Statistics Austria	IQ90 – IQ20					1	
Population aged 6–14 at begin of quarter	Statistics Austria	IQ90 – IQ20					1	
Salaried employees and civil servants aged 15–64, men, in percent of population of the same age group	Federation of Austrian Social Security Institutions	1M95 – 1M21					1	
Salaried employees and civil servants aged 15–64, women, in percent of population of the same age group	Federation of Austrian Social Security Institutions	1M95 – 1M21					1	
Workers aged 15–64, men, in percent of population of the same age group	Federation of Austrian Social Security Institutions	1M95 – 1M21					1	
Workers aged 15–64, women, in percent of population of the same age group	Federation of Austrian Social Security Institutions	1M95 – 1M21					1	
Domestic gas consumption	E-Control	1M00 – 12M20						1
Industrial production—manufacture of products of wood, cork, straw, and plaiting materials, 2015 = 100	Statistics Austria	1M96 – 12M20						1
Market consumption of fuels (diesel, regular gasoline, premium gasoline)	Federal Ministry of Climate Action, Environment, Energy, Mobility, Innovation and Technology	1M90 – 9M20						1
Net exports of cork and wood, Euro	Statistics Austria	1M06 – 11M20						1
Net exports of crude materials and fuels, Euro	Statistics Austria	1M06 – 11M20						1
Net exports of hides and skins, Euro	Statistics Austria	1M06 – 11M20						1
Net exports of mineral crude materials, Euro	Statistics Austria	1M06 – 11M20						1
Net exports of other crude materials, Euro	Statistics Austria	1M06 – 11M20						1
Net exports of solid fuels, Euro	Statistics Austria	1M06 – 11M20						1
Net exports of textile crude materials, Euro	Statistics Austria	1M06 – 11M20						1
Wood harvest	WIFO, BMLFUW	IQ95 – IVQ20						1

High-frequency variables are presented in descending order of the number of models for which they are used. Within each group they are sorted alphabetically

## Footnotes

1 These can be extended with historical data from the ESA1995.

2 The root mean squared error is the square root of the average of the squared differences between the predicted and the observed values. In the case of SDG 01_41 and SDG 05_40 the short sample size of the series itself, or of the input series allows an out-of-sample analysis only between 2016–2019 or 2017–2019, respectively.

## Code availability

Available on request.
